# Editorial: RNA Biology in Cardiovascular Disease

**DOI:** 10.3389/fgene.2021.775091

**Published:** 2021-10-12

**Authors:** Maarten M. G. van den Hoogenhof, Hamid El Azzouzi, Abdelaziz Beqqali

**Affiliations:** ^1^ Institute of Experimental Cardiology, Heidelberg University Clinic, Heidelberg, Germany; ^2^ Partner Site Heidelberg, German Center for Cardiovascular Research (DZHK), Heidelberg, Germany; ^3^ Department of Molecular Genetics, Erasmus University Medical Center, Rotterdam, Netherlands; ^4^ Centre for Cardiovascular Science, The Queen’s Medical Research Institute, The University of Edinburgh, Edinburgh, United Kingdom

**Keywords:** RNA biology, heart, RNA splicing, cardiovasclar disease, non-coding RNAs

In the last years, RNA biology has gained significant attention in cardiovascular science. The rise of next-generation sequencing has not only uncovered various previously unknown types of RNA, but also a much greater extent of the function(s) of RNA than previously realized. For example, multiple pivotal cardiac splicing factors, such as RBM20 and RBM24, have recently been identified (Guo et al., 2012; Yang et al., 2014); the functional role of RNA modifications such as m6A are started to be elucidated (Dorn et al., 2019), and the roles of noncoding RNAs (ncRNAs), such as lincRNAs (Hobuss et al., 2019) and circRNAs (Aufiero et al., 2019), are being unravelled. Heart disease remains one of the major health problems in the world, and changes in RNA biology can both be a cause and a consequence of disease (van den Hoogenhof et al., 2016). These new insights in RNA biology underline the need to study the diverse roles for RNA in the healthy and diseased heart more comprehensively. In this Research Topic, we focus on novel roles of ncRNAs, but also review new insights in techniques and processes related to RNA biology in the heart.

One of the major advances in RNA sequencing, is single-cell RNA sequencing (as opposed to bulk RNA sequencing). Marin-Sedeño et al. review what single-cell RNA sequencing has meant for the cardiovascular field, more specifically how it has been used to shape the cardiac cellular landscape both in healthy and diseased hearts. In addition, they discuss drawbacks and future perspectives of this relatively new and powerful tool. Another advantage of the advance of next generation sequencing is the increased availability of transcriptomic data. This makes it possible to (re)analyze published datasets with new questions in mind, and use this as a starting point for new studies. Nie et al. used published transcriptomic data to gain insight in abdominal artery aneurisma (AAA), a condition that kills approximately 13.000 people every year. Their study showed that AAA samples could be distinguished from healthy samples by the infiltration of immune cells, and they identified 5 key immunological genes, SSTR1, GPER1, CCR10, PI3, and MAP2K1, that may be involved in AAA. Yan et al. used publicly available data to provide a weighted gene correlation network analysis of immune cells in atrial tissue of AF patients as compared to non-AF. They identified and confirmed high atrial expression of three genes (CTSS, CSF2RB and NCF2) which are strongly associated with AF. The authors propose these three candidate genes may play an important role in the development of AF and may serve as potential therapeutic targets for AF treatment. However, further in-depth studies are needed to unravel what role these three genes have in the molecular mechanisms underlying AF. Wang et al. used publicly available datasets to identify miRNAs and their targets in aortic dissection. They identified miR-193a-3p and its target ACTG2 to regulate vascular smooth muscle cell proliferation and migration and phenotypic switching. Taken together, they provide a novel molecular pathway in the development of aortic dissection that has the potential to be therapeutically targeted.

Abu-Halima et al. have characterized the blood miRNA profile of univentricular heart patients with and without Fontan palliation using a large panel of miRNAs for initial screening in order to identify those that may indicate advanced liver fibrosis/cirrhosis and thus might have clinical as well as prognostic impact in this cohort of patients. This screen led to the identification of microRNA-29b/c-3p and the authors propose that this miR represents an independent predictor of advanced liver fibrosis/cirrhosis and may be used in the risk assessment of these patients. Pham et al. identified a novel LncRNA, which they termed Aerrie, in HUVECs exposed to shear stress. They found that Aerrie is expressed in the endothelium, where it regulates endothelial function through binding with YBX1 and controlling DNA damage repair. Since Aerrie is upregulated during aging, this suggests that Aerrie is crucial to maintain proper endothelial cell function during life. Another important subclass of non-coding RNAs are circular RNAs (circRNAs), which are characterized by a stable structure without free ends. In the work of Xu et al., after circRNA-sequencing of atherosclerotic mouse aortas, a circRNA called circDENND1B was shown to have an important role in modulating atherosclerosis through regulation of cholesterol efflux. By acting as a sponge for miR-17-5p, circDENND1B induced an increase in ATP binding cassette subfamily A member 1 (Abca1) expression and thus promoting cholesterol efflux. Moreover, circDENND1B could alleviate foam cell formation, and is involved in modulating inflammation enhancing the antiatherosclerosis effect of IL-1b mAb in mice. While this work elegantly provides new insights into the interaction between inflammation and cholesterol transport, the therapeutic potential of circDENND1B needs further exploration.

While much of the RNA research of the last decade centred around its non-coding forms and the numerous roles these ncRNAs have in cellular physiology, the classical coding role of RNA in the heart is still fascinatingly complex. Duran et al. showcased this in their effort to sum-up the tremendous insights into CaMKII dependent signalling. Encompassing 12 subunits, the oligomerization of this holoenzyme is multifaceted with the existence of different splice variants adding to the complexity. Importantly, the authors highlight that lesser expression doesn’t mean lesser importance and that next to the four isoforms described in the review other isoforms that are less abundant in the heart might have surprisingly important spatio-temporal effects during disease. Obviously, more work is needed to understand the role of the different splice variants that code this vital signalling complex. What is less obvious but important to realize, is that studying ncRNAs that target a complex without understanding this complex’ true composition is less fruitful. Three-dimensional genome organization has been extensively studied and has emerged as an important regulator of gene expression. In his article, Bertero discusses the possibility that interchromosomal genome architecture can be regulated by RNA biogenesis, and uses the example of RNA polymerase II clusters and foci of the cardiac-specific splicing regulator RBM20 to show the existence and functionality of *trans*-interacting chromatin domains. With this, the author extends on an exciting but not completely understood paradigm of gene regulation, and proposes an experimental framework with which to test this hypothesis. And lastly, an elegant study by Boileau et al. shows a comprehensive integrated analysis of RNA-sequencing and Ribo-sequencing datasets in the same heart samples under physiological vs pathological remodeling. The authors have identified changes in transcriptome and translatome which are specific to physiological vs pathological changes using their hypertrophy mouse models. They found that transcriptome networks are only partially reproducible at the translatome level, providing further evidence of the importance of post-transcriptional control at the level of translation. Their results provide novel insights into the complexity of the organisation of *in vivo* cardiac regulatory networks.

The diverse collection of articles in this Research Topic shows that RNA biology is a vast and vibrant field of study in cardiovascular biology, and the immense advances in next-generation sequencing and its analysis methods will lead to new and exciting insights into the heart.
